# Deep learning–based MR‐to‐CT synthesis: The influence of varying gradient echo–based MR images as input channels

**DOI:** 10.1002/mrm.28008

**Published:** 2019-10-08

**Authors:** Mateusz C. Florkow, Frank Zijlstra, Koen Willemsen, Matteo Maspero, Cornelis A. T. van den Berg, Linda G. W. Kerkmeijer, René M. Castelein, Harrie Weinans, Max A. Viergever, Marijn van Stralen, Peter R. Seevinck

**Affiliations:** ^1^ Image Sciences Institute University Medical Center Utrecht Utrecht Netherlands; ^2^ Department of Orthopedics University Medical Center Utrecht Utrecht Netherlands; ^3^ Department of Radiotherapy Division of Imaging & Oncology University Medical Center Utrecht Utrecht Netherlands; ^4^ Computational Imaging Group for MR diagnostics & Therapy Center for Image Sciences University Medical Center Utrecht Utrecht Netherlands; ^5^ MRIguidance B.V Utrecht Netherlands

**Keywords:** deep learning, gradient echo, MR contrasts, synthetic CT

## Abstract

**Purpose:**

To study the influence of gradient echo–based contrasts as input channels to a 3D patch‐based neural network trained for synthetic CT (sCT) generation in canine and human populations.

**Methods:**

Magnetic resonance images and CT scans of human and canine pelvic regions were acquired and paired using nonrigid registration. Magnitude MR images and Dixon reconstructed water, fat, in‐phase and opposed‐phase images were obtained from a single T_1_‐weighted multi‐echo gradient‐echo acquisition. From this set, 6 input configurations were defined, each containing 1 to 4 MR images regarded as input channels. For each configuration, a UNet‐derived deep learning model was trained for synthetic CT generation. Reconstructed Hounsfield unit maps were evaluated with peak SNR, mean absolute error, and mean error. Dice similarity coefficient and surface distance maps assessed the geometric fidelity of bones. Repeatability was estimated by replicating the training up to 10 times.

**Results:**

Seventeen canines and 23 human subjects were included in the study. Performance and repeatability of single‐channel models were dependent on the TE‐related water–fat interference with variations of up to 17% in mean absolute error, and variations of up to 28% specifically in bones. Repeatability, Dice similarity coefficient, and mean absolute error were statistically significantly better in multichannel models with mean absolute error ranging from 33 to 40 Hounsfield units in humans and from 35 to 47 Hounsfield units in canines.

**Conclusion:**

Significant differences in performance and robustness of deep learning models for synthetic CT generation were observed depending on the input. In‐phase images outperformed opposed‐phase images, and Dixon reconstructed multichannel inputs outperformed single‐channel inputs.

## 1 INTRODUCTION

The combined use of MRI and CT has proven useful in radiotherapy planning,^1^ orthopedics,^2^, ^3^ and for PET/MR attenuation correction.^4^ Magnetic resonance imaging provides images with a high soft‐tissue contrast required for soft‐tissue delineation. Computed tomography scans provide tissue radiodensity maps—a property expressed in Hounsfield units (HU)—that poorly discriminate soft tissues, but are perfectly suited to visualize osseous tissues and enable the calculation of dose and attenuation coefficient maps. Despite having benefits, the consecutive imaging sessions increase the patient burden and introduce complex workflows with error‐prone intermodality registration and extra costs, which motivate research into MR‐only workflows. In such workflows, HU maps are reconstructed based on MR information. No intermodality registration is required, and there is a perfect voxel‐wise correspondence among all of the MR‐generated information. The main challenge resides in the accurate retrieval of HU values from MR images that do not directly measure radiodensity.

Many different techniques to generate a CT surrogate, referred to as synthetic CT (sCT), have been developed in recent years, primarily in the pelvis and head for radiotherapy treatment planning^5^, ^6^, ^7^ and for PET/MR purposes.^8^, ^9^, ^10^, ^11^ A recent study^12^ has also been proposed in the lower arm for orthopedic purposes. These techniques can roughly be categorized as atlas‐based^9^, ^13^ methods or voxel‐based^14^, ^15^, ^16^, ^17^, ^18^, ^19^ methods, which include deep learning methods. They use different images as inputs, including acquired T_1_‐weighted,^9^, ^16^ T_2_‐weighted,^13^, ^14^ ultrashort TE^17^, ^18^ or zero‐TE^8^ (ZTE) images, reconstructed Dixon^17^ images, or combinations of these.^15^, ^19^ Dixon‐based techniques have been used, as they facilitate water and fat separation, simplifying soft‐tissue HU assignment. Proton density–weighted ZTE images depict cortical bone with a specific low signal, potentially simplifying its identification.^20^, ^21^, ^22^

In parallel to the use of specialized and combined MR images, processing techniques have also expanded to involve statistical^17^ and machine learning models,^14^ with recent advances in deep learning.^6^, ^10^, ^12^, ^23^, ^24^, ^25^, ^26^, ^27^, ^28^, ^29^, ^30^, ^31^ Recent studies^23^, ^25^, ^31^ have shown that deep learning–based models generate equivalent or better sCTs than more conventional methods. Particularly encouraging results were obtained for radiotherapy treatment planning purposes^6^, ^7^ and PET/MR^10^, ^11^, ^24^, ^29^ while using automated workflows. Most studies using deep learning exploit only single images obtained from a T_1_‐weighted gradient‐echo acquisition.^24^, ^25^, ^26^, ^27^ Recently, the information content of gradient‐echo images was further exploited by using Dixon reconstructions from multi‐echo gradient‐echo acquisitions as inputs to a neural network.^6^, ^11^ Furthermore, proton density–weighted ZTE images have been used in the last few years^10^, ^29^ to facilitate the discrimination between cortical bone and air, despite a potentially limited benefit.^32^

Intuitively, the performance of deep learning models is influenced by the data by which they are trained and evaluated. We hypothesize that combinations of MR images obtained from gradient‐echo experiments could provide the neural network with additional information about tissue composition including proton density, water and fat fractions, as well as magnetic properties such as relaxation constants (T_1_, $T_{2}^{*}$) and susceptibility.^33^ These properties may be particularly useful for the task of mapping MR data into HU values. However, the exact impact of these combinations of MR images, referred to as MR input configurations for the remainder of this paper, is unknown.

Therefore, in this study, we trained deep learning–based models using several MR input configurations with varied information about these properties. Each input configuration contained 1 or several images acquired or reconstructed from a T_1_‐weighted multi‐echo gradient‐echo sequence. Synthetic CTs generated by each model were evaluated using voxel‐wise and image‐based similarity metrics. Models were trained independently on 2 data sets to assert the robustness of the results to changes in acquisition parameters and physiological variations. Replications of the training assessed its repeatability and the statistical significance of the differences seen in the metrics.

## 2 METHODS

### 2.1 Data collection

The reported study was performed on images of the pelvic region of canine (ex vivo) and human (in vivo) populations. The canine population provides a natural animal model for osteoarthritis^34^ and hip dysplasia.^35^ For conservation purposes, the dogs were frozen and removed from the freezer 2 days before the scanning.

The canine data set consisted of 18 domestic dogs (5 males and 13 females) that deceased of natural causes and were admitted to the veterinary department for scientific purposes. The MR images were acquired in a fixed supine position in a 1.5T scanner (Ingenia; Philips Healthcare, Best, Netherlands) using a 3D RF spoiled T_1_‐weighted multiple gradient‐echo (T_1_w‐MGE) sequence. The following parameters were used during the acquisition: TE = 2.03 ms/4.28 ms, TR = 6.7 ms, flip angle = 20°, FOV = 270 × 270 × 120 mm^3^, acquired voxel size = 1 × 1 × 1.34 mm^3^, reconstructed voxel size = 0.6 × 0.6 × 0.67 mm^3^, bandwidth = 541 Hz/px, and acquisition time = 4 minutes 38 seconds.

The human data set consisted of 27 male patients diagnosed with prostate cancer who underwent intensity‐modulated radiotherapy. The T_1_w‐MGE MR images were acquired in a head‐first supine position on a flat table in a 3T scanner (Ingenia; Philips Healthcare). A coil bridge and a knee wedge guaranteed similar positioning between MRI and CT scanning. The images were acquired in 2 minutes 38 seconds with TR = 6.5 ms, TE = 2.1 ms/3.5 ms/4.8 ms, bandwidth = 1122 Hz/pixel, FOV = 435 × 435 × 160 mm^3^, flip angle = 10º, acquired voxel size = 1.2 × 1.2 × 2 mm^3^, and reconstructed voxel size = 0.97 × 0.97 × 1 mm^3^.

For both acquisitions, 1 echo was acquired almost in phase (aIP—first echo for the human data set acquired at 3 T and second echo for the canine data set acquired at 1.5 T) and 1 almost opposed phase (aOP—second echo for the human data set, first echo for the canine data set). A Dixon reconstruction^36^, ^37^ was performed on the scanner to obtain in‐phase (IP), opposed phase (OP), fat only (F), and water only (W) images for all subjects.

**Figure 1:**
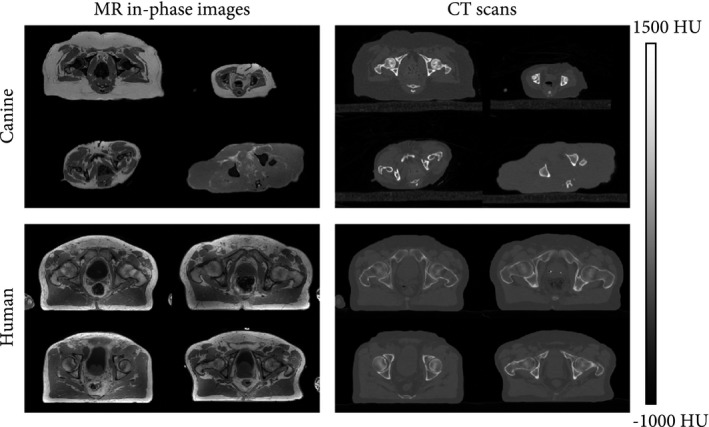
Transverse slices of the canine and human data sets (paired in‐phase MR images and CT scans). As compared with the human data set, the canine data set showed substantial intersubject variability in morphology (e.g., shape, size). Abbreviation: HU, Hounsfield unit

The CT scans (Brilliance CT Big Bore; Philips Healthcare) were acquired with a slice spacing of 0.7 mm and a pixel spacing ranging from 0.3 mm to 0.7 mm in the canine data set. Tube current ranged from 30 mA to 66 mA. For the human subjects, the CT slice spacing was 3 mm, and the pixel spacing ranged from 0.8 mm to 1.12 mm. Tube current ranged from 62 mA to 154 mA. For both data sets, tube voltage was 120 kV. Less than an hour separated the acquisitions on both modalities. Figure 1 provides samples of both data sets.

We excluded scans that contained foreign metallic body inside or in the surroundings of the FOV, as they could cause artifacts in either modality. All images were acquired in accordance with the regulations from the local ethical committee.

By working on these 2 data sets, we confirmed the robustness of the findings to variations in acquisition parameters and physiology. In addition, the in‐phase and opposed‐phase images were acquired at different TEs on both data sets, providing insight on the effect of $T_{2}^{*}$ decay on the model.

### 2.2 Input configurations

**Figure 2:**
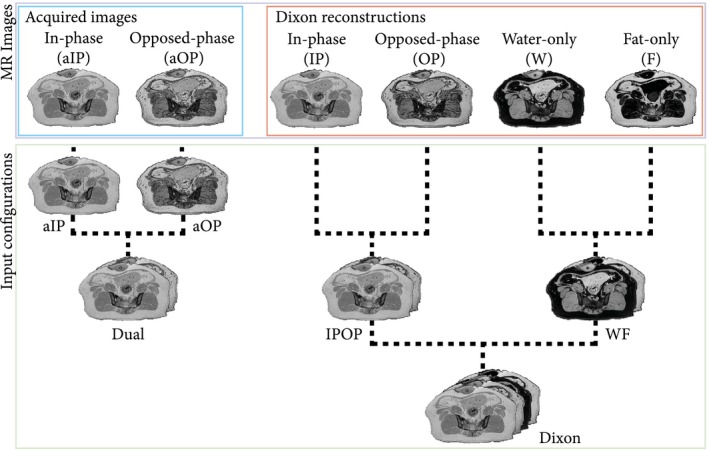
Description of how MR images from the T_1_‐weighted multiple gradient‐echo (T1w‐MGE) were combined to form all 6 input configurations. Images were concatenated along the channel dimension, resulting in single‐channel input configurations (almost in phase [aIP] and almost opposed phase [aOP]) or multichannel input configurations (acquired images [Dual], Dixon reconstructed images [IPOP], water–fat decomposition [WF], and Dixon)

Six input configurations were investigated in the study, each containing 1, 2, or 4 channels. Here a channel refers to an MR image obtained from the single T1w‐MGE acquisition. In particular, we investigated the 2 first acquired echoes (aIP and aOP) and the Dixon reconstructed IP, OP, W, and F images. These images were combined into 6 input configurations called aIP, aOP, Dual, IPOP, WF, and Dixon, which are defined in Figure 2. The input configurations were designed to study the influence of several properties of the tissues including $T_{2}^{*}$ weighting (aIP and aOP), water–fat interference (aIP and aOP), and water–fat decomposition (WF), and to compare the acquired images (Dual) against Dixon reconstructed images (IPOP).

**Figure 3:**
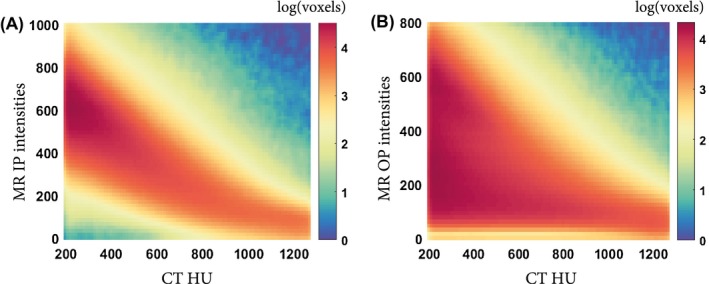
Two‐dimensional histograms between MRI intensities and CT Hounsfield units for voxels corresponding to cortical bone (HU > 200). The correlation between bone voxel intensities in CT scans and MR images is stronger in in‐phase (IP) images (A) than in opposed phase (OP) images (B)

Because each image within an input was considered as a channel, input configurations containing more than 1 image are referred to as multichannel as opposed to single‐channel input configurations. For our statistical comparison, we preferred the use of the aIP configuration as a reference, because it is a single‐channel configuration with a higher correlation between MR intensities and CT HU than the aOP configuration, as shown in Figure 3.

### 2.3 Experimental setup

#### 2.3.1 Constraints

To perform a fair comparative study, a single network architecture was used to process the heterogeneous data sets and the variable input configurations. Notably, the choices defining the neural network architecture and its hyperparameters were constrained by 2 requirements: the neural network needed to (1) handle inputs containing different numbers of images without increasing the number of trainable parameters (fixed complexity) and to (2) make repeatable predictions in order to identify input‐related changes easily in the reconstructed sCTs. The remainder of this section describes the neural network as it was implemented.

#### 2.3.2 Architecture

To generate sCT images from MR inputs, we used a patch‐based convolutional neural network: a 3D extension of the widely used U‐Net.^38^, ^39^ This architecture took as input 4D MR images with 3 spatial dimensions and a channel dimension. The size of the patches was C × 24 × 24 × 24 voxels, where C is the number of channels in the input configuration. Our implementation included 3 × 3 × 3 convolution layers, 2 × 2 × 2 max pooling layers, instance normalization,^40^ and rectified linear unit activation layers, resulting in about 4 million trainable parameters. A Glorot uniform initialization^41^ was used to initialize the kernels. Upsampling was implemented using nearest‐neighbor interpolation to avoid the checkerboard artifact that can occur in image generation tasks.^42^ The design of this architecture did not favor inputs with multiple channels, as all channels were combined in the first layer, which increased the number of trainable parameters by less than 0.1% between inputs containing 1 and 4 channels. The network was implemented using Keras 2.1.3 with a Tensorflow 1.7 backend. The training and evaluation of the model were performed with 2 GeForce GTX 1080 Ti (NVIDIA, Santa Clara, CA) GPUs.

#### 2.3.3 Training

An experiment consisted of the training of a neural network with 1 input configuration. The canine and human data sets being distinct, models were trained independently on each population. For each data set, a 3‐fold cross‐validation procedure was applied to synthetize sCTs. Note that models trained on 1 data set were only evaluated on that data set.

Models were trained with a Nadam^43^ optimizer whose objective was to minimize the L_1_ loss between the CT and the sCT. The learning rate was constant and set at 10^−4^. With the L_1_ loss function, the optimization process was robust to outliers produced by noise, artifacts, or by the imperfect matching between MR and CT. It also resulted in sharper images than the L_2_ norm.^44^ Patches used to perform an optimization step were randomly extracted from the MR images but were balanced between soft tissues and bone by using a weighted probability map that resulted in an equal sampling of bone and nonbone voxels. Because 6 input configurations were tested, 6 independent models were optimized per fold. Parameters defined for the optimization were kept identical for the training of all models.

#### 2.3.4 Replications

Because the training of the neural network contained random factors, the training of each model was replicated multiple times. This evaluated the repeatability of sCT generation and corroborated the statistical significance of the findings. Experiments were repeated 5 times on the human data set and 10 times on the canine data set, because the latter presented a larger anatomical variability (Figure 1).

### 2.4 Data processing

#### 2.4.1 Image preprocessing

The CT scans were registered to the magnitude of the aIP image using the Elastix registration toolbox^45^ to create a voxel‐wise MRI‐CT matching for training and evaluation. The registration was a composition of a rigid Euler transform and a nonrigid B‐spline transform, both optimized with an adaptive stochastic gradient descent procedure with mutual information as the similarity metric. A rigidity penalty^46^ was applied to the entire volume during deformable registration. In addition, CT scans were resampled to match the resolution of MR images using third‐order B‐spline interpolation. The parameter files used for the registration can be found at http://elastix.bigr.nl/wiki/index.php/Par0059. No registration was needed between input channels, as they contained MR images or Dixon reconstructions obtained from a single T1w‐MGE sequence.

To facilitate the training of each model, a per‐subject linear normalization was applied on MR images and CT scans. The normalization mapped intensities to [−1; 1] using

(1) $$I_{\text{norm}}=\left(\frac{I-\text{shift}}{\text{scale}}\right)*2-1.$$

For the MR image, the shift and scale were derived from minimal and maximum intensities. For the CT, these values were constant to preserve HU quantitative nature and were set to −1000 for the shift and 4000 for the scale.

#### 2.4.2 Masking

To include only relevant anatomy and to perform a tissue‐specific evaluation, binary masks were automatically derived from the MR in‐phase images and CT scans by application of thresholding and mask filling. For both modalities, the average intensity of the image was used as a threshold value. The intersection of MR and CT body masks isolated the volume of interest from the background. Bone voxels in the CT scan whose intensity was greater than 200 HU and that were within the volume of interest were defined as bone.

### 2.5 Evaluation

#### 2.5.1 Synthetic CT synthesis

Prediction of the sCT was based on the extraction of overlapping MR patches followed by a weighted mean fusion. In total, most voxels were predicted 72 times.

#### 2.5.2 Metrics

The similarity between the CT and sCT was assessed using 5 metrics: mean error (ME), mean absolute error (MAE), Dice similarity coefficient (DSC), surface distance, and peak SNR (PSNR). The ME and MAE are voxel‐wise differences commonly reported in research for sCT generation and reflecting the sCT fidelity in radiodensity reconstruction, which is particularly useful for radiotherapy planning and PET/MR reconstruction. They were computed on the entire body contour (MAE_body_) and exclusively on the bone (MAE_bone_) using the aforementioned masks. The PSNR approximates human perception of reconstruction quality and was defined as

(2) $$\text{PSNR}=10\log_{10}\left(\frac{4095^{2}}{\text{1}/N\sum_{i=1}^{N}{\left(I_{\text{CT}}\left(i\right)-I_{\text{sCT}}\left(i\right)\right)}^{2}}\right).$$

To estimate the degree of misclassification and the geometric integrity of the bone, DSC^47^ was computed with regard to bone. Being a global measure of overlap, DSC does not quantify local discrepancies between surfaces useful for orthopedics. Consequently, surface distance maps were computed to evaluate local surface dissimilarities by measuring the bilateral distance^48^ between 2 surfaces. Bone segmentations used to compute the surface distance maps were obtained by thresholding the CT and sCT at 200 HU and by automatically excluding nonosseous, unconnected components. The structural similarity index between the CT and sCT was also measured and is reported in Supporting Information Table S1.

Each measurement had 3 dimensions: a subject dimension *s*, an input configuration *c*, and a replicate dimension *r*. To evaluate the differences between input configurations within 1 subject, metrics were averaged across replicates *r* such that

(3) $${\bar{\text{Metric}}}^{R}\left(s,\;c\right)=\frac{1}{\#\text{replicates}}\sum_{r=1}^{\#\text{replicates}}\text{Metric}\left(r,\;s,\;c\right).$$

To gain a general overview of the performance of an input configuration across a population, metrics were averaged across replicates and subjects such that

(4) $${\bar{\bar{\text{Metric}}}}^{\text{RS}}\left(c\right)=\frac{1}{\#\text{subjects}}\sum_{s=1}^{\#\text{subjects}}{\bar{\text{Metric}}}^{R}\left(s,\;c\right).$$

The SD σ(Metric) was computed across the (human or canine) population as follows:

(5) $$\sigma^{\text{Metric}}\left(c\right)=\sqrt{\frac{1}{\#\text{subjects}}\sum_{s=1}^{\#\text{subjects}}{\left({\bar{\text{Metric}}}^{R}\left(s,\;c\right)-{\bar{\bar{\text{Metric}}}}^{\text{RS}}\left(c\right)\right)}^{2}}.$$

For the remainder of the paper, the input configuration *c* will be implied if not present.

#### 2.5.3 Repeatability

The repeatability per input configuration was assessed by calculating the standard deviation of ${\text{MAE}}_{\text{body}}\left(r,\;s\right)$ averaged across subjects, as follows:

(6) $$\text{Repeatability}=\frac{1}{\#\text{subjects}}\sum_{s=1}^{\#\text{subjects}}\sqrt{\frac{1}{\#\text{replicates}}\sum_{r=1}^{\#\text{replicates}}{\left({\text{MAE}}_{\text{body}}\left(r,\;s\right)-{\bar{{\text{MAE}}_{\text{body}}}}^{R}\left(s\right)\right)}^{2}}.$$

#### 2.5.4 Statistical analysis

We tested the significance of the differences observed between sCTs using a repeated‐measure analysis of variance. The null hypothesis assumed that all models achieved the same average MAE_body_, regardless of their input configuration. Because the variance of MAE_body_ was model‐dependent, a Greenhouse‐Geisser sphericity correction^49^ was applied to meet the homogeneity of variance assumption required for the test. When the repeated‐measure analysis of variance rejected the null hypothesis at a 95% confidence level, the sCT generated from different input configurations was compared pairwise using post hoc Student’s t‐tests with Bonferroni correction for repeated pairwise comparisons. Statistical tests were performed on combined results from both data sets on IBM SPSS Statistics 23 using, for each subject, the MAE_body_ averaged across replicates $\left({\bar{{\text{MAE}}_{\text{body}}}}^{R}\left(s\right)\right)$. For all statistical tests, *P* < .05 was considered to be statistically significant.

## 3 RESULTS

In the human data set, 24 of 27 subjects met the inclusion criteria of the study. All dogs were eligible except for 1 (17 of 18), whose MR acquisition protocol did not follow the study design format. The training set in each cross‐validation fold contained 16 subjects for the human models and 11 for the canine models. The remainder of this section presents the results of all 3 folds of the cross‐validation for both data sets.

### 3.1 Per subject results

**Figure 4:**
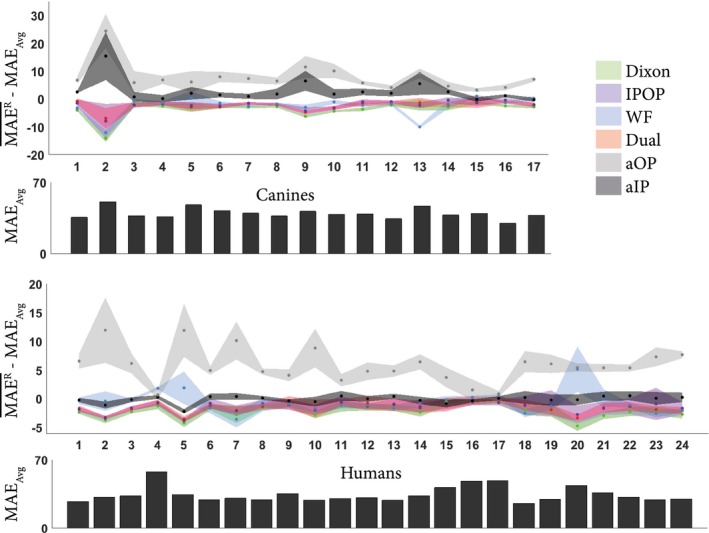
Variation of mean absolute error (MAE) per subject averaged across the repeated experiments ($\bar{{\text{MAE}}^{R}}$) for each input configuration. The shading represents the SD of MAE_body_ for each subject across replicates. To focus on variations between input configurations and remove intersubject variability, measurements were zero‐centered per subject: The average MAE across replicates and input configurations (MAE_Avg_) was subtracted from $\bar{{\text{MAE}}^{R}}$. Therefore, negative values for the relative MAE indicate better performance. For completeness, MAE_Avg_ per subject is present in the bar plot, indicating the intersubject variability

Figure 4 presents the average MAE_body_ across replicates (${\bar{{\text{MAE}}_{\text{body}}}}^{R}\left(s\right)$) obtained for each model. From this figure, it can be observed that (1) within single‐channel models, aIP‐based models always outperformed aOP‐based models, (2) all multichannel models but WF were equivalent to or outperformed single‐channel models, and (3) differences were small among multichannel models. These behaviors were observed in all subjects from both data sets.

### 3.2 Per model results

Jointly performed on the canine and human data sets, the repeated‐measure analysis of variance demonstrated a significant difference between models (*P* < 10^−12^), which was categorized into 3 levels using post hoc t‐tests.

First, within single‐channel models, ${\bar{\bar{{\text{MAE}}_{\text{body}}}}}^{\text{RS}}\left(\text{aIP}\right)$, the MAE_body_ averaged across replicates and subjects for the aIP input configuration, was lower than ${\bar{\bar{{\text{MAE}}_{\text{body}}}}}^{\text{RS}}\left(\text{aOP}\right)$ by 5.5 HU, which renders the aIP input configuration favorable over aOP (corrected t‐test, *P* < 10^−56^). The value of ${\bar{\bar{{\text{MAE}}_{\text{body}}}}}^{\text{RS}}\left(\text{aOP}\right)$ was 12% higher for the canine population and 17% higher for the human population, as indicated in Table 1, which reports the average performance obtained per input configuration and data set (${\bar{\bar{∙}}}^{\text{RS}}$).

**Table 1:** Performance (± *σ*) obtained for each model per data set, as measured by mean absolute error (MAE), mean error (ME), Dice similarity coefficient (DSC), surface distance, and peak SNR (PSNR) averaged across replicates and subjects

Population	Input	MAE_body_ (HU)	ME_body_ (HU)	MAE_bone_ (HU)	DSC_bone_ (1)	Surface distance (mm)	PSNR (dB)
Human	aIP	34.1 ± 7.9	−1.2 ± 4.6	123 ± 54	0.81 ± 0.11	0.45 ± 0.10	36.1 ± 2.3
	aOP	40.4 ± 6.7	−0.0 ± 9.2	158 ± 42	0.78 ± 0.10	0.49 ± 0.10	34.7 ± 1.7
	Dual	32.7 ± 8.3	−2.0 ± 4.3	117 ± 56	0.83 ± 0.11	0.42 ± 0.05	36.4 ± 2.4
	IPOP	32.7 ± 8.1	−1.4 ± 4.6	116 ± 56	0.83 ± 0.11	0.42 ± 0.05	36.4 ± 2.4
	WF	33.6 ± 9.2	−1.2 ± 5.1	120 ± 58	0.82 ± 0.11	0.42 ± 0.16	35.9 ± 2.5
	Dixon	32.1 ± 8.3	−1.5 ± 4.9	114 ± 56	0.83 ± 0.11	0.40 ± 0.03	36.5 ± 2.5
Canine	aIP	42.2 ± 8.1	−0.9 ±14.1	144 ± 42	0.91 ± 0.03	0.38 ± 0.14	35.1 ± 1.6
	aOP	47.2 ± 9.1	−3.8 ±16.3	169 ± 37	0.89 ± 0.04	0.57 ± 0.28	34.1 ± 1.5
	Dual	37.1 ± 4.3	−0.4 ± 7.3	132 ± 36	0.92 ± 0.03	0.38 ± 0.14	35.9 ± 1.5
	IPOP	37.2 ± 4.1	−0.1 ± 6.8	134 ± 36	0.92 ± 0.03	0.37 ± 0.14	35.8 ± 1.5
	WF	36.9 ± 4.1	−0.6 ± 6.6	141 ± 34	0.92 ± 0.03	0.37 ± 0.14	35.4 ± 1.7
	Dixon	35.8 ± 4.2	−1.4 ± 6.8	131 ± 34	0.92 ± 0.04	0.38 ± 0.14	36.1 ± 1.7

Second, aIP‐based models, which have the lowest ${\bar{\bar{{\text{MAE}}_{\text{body}}}}}^{\text{RS}}$ of single‐channel models, were always outperformed by multichannel models with differences of up to 15% for the canine population and almost 6% for the human population (Table 1). On average, when compared with aIP‐based models, the difference between the sCT and the CT was 3.8 HU lower for Dixon models (corrected t‐test, *P* < 5.10^−18^), 2.8 HU lower for IPOP and Dual models (corrected t‐test, *P* < 5.10^−16^), and 2.5 HU lower for WF models (corrected t‐test, *P* < 10^−7^). In the heterogeneous canine data set, single‐channel models were also less robust with higher SD than multichannel models, which reflected higher intersubject variations.

Third, within multichannel input configurations, IPOP, WF, and Dual models obtained similar results but were outperformed by Dixon models by up to 1.3 HU difference (corrected t‐test, *P* < 5.10^−10^). Although statistically significant, this difference was not observed in all subjects. In particular, the WF model strongly outperformed the Dixon model once (Figure 4, canine data set, subject 13) in a subject that presented soft‐tissue lesions.

Apart from aOP, all input configurations achieved a repeatability under 1 HU for the humans and under 2 HU for the canines, as given in Table 2. This high repeatability corroborates the statistical significance of the aforementioned differences.

**Table 2:** Repeatability (± *σ*) obtained for each model per data set, averaged across replicates and subjects

Population	Input	Repeatability (HU)
Human	aIP	0.6 ± 0.3
	aOP	1.6 ± 1.3
	Dual	0.7 ± 0.3
	IPOP	1.0 ± 0.6
	WF	0.9 ± 0.9
	Dixon	0.7 ± 0.3
Canine	aIP	1.9 ± 2.0
	aOP	2.1 ± 1.5
	Dual	1.1 ± 0.8
	IPOP	1.2 ± 1.3
	WF	0.8 ± 0.5
	Dixon	0.7 ± 0.5

### 3.3 Bone reconstruction

Regardless of the data set and input configuration, MAE_bone_ was almost 4 times higher than MAE_body_, with most ${\bar{\bar{{\text{MAE}}_{\text{bone}}}}}^{\text{RS}}$ ranging between 114 HU and 123 HU, and ${\bar{\bar{{\text{MAE}}_{\text{body}}}}}^{\text{RS}}$ between 32 HU and 34 HU in the human data set (Table 1).

**Figure 5:**
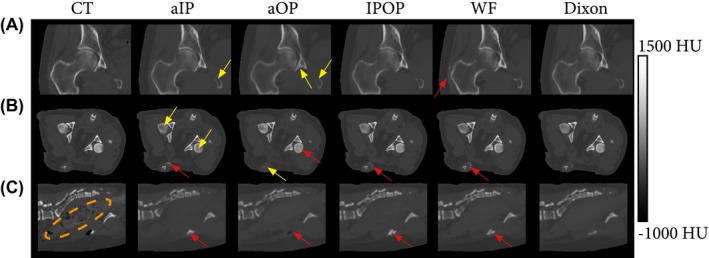
Computed tomography and synthesized CTs (sCTs) generated per input configurations for 3 subjects. The Dual configuration was omitted, as it was very similar to IPOP. A, Coronal view of the right femoral head and acetabulum of a human patient (subject 1). B, Transversal view of the pelvic anatomy of a canine subject (subject 1). C, Sagittal view of the spine of a canine subject (subject 12). Yellow arrows indicate hypo‐intense bone regions; red arrows indicate hyperintense regions; and the orange encircled area indicates the bowels that do not contain any air pockets on the sCTs

Figure 5 shows the sCTs obtained by each model for 3 subjects. Although reflected by global metrics, the qualitative differences between sCTs were primarily local and especially observed in osseous structures such as the baculum or vertebrae.

**Figure 6:**
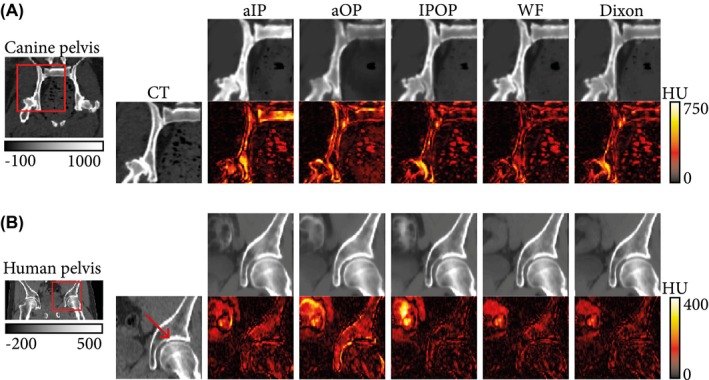
Comparison of sCTs generated by the input configurations for canine (A) and human (B) subjects with a focus on bone. The Dual input configuration was omitted, as it was very similar to IPOP. A region defined by the red square was enlarged and its window level adapted to highlight bone structures. Errors maps show the absolute errors between the sCTs and the CT. For the human patient, the red arrow shows a sclerotic (hyperintense) region of the acetabulum, called the acetabular sourcil, which cannot be easily distinguished from the rest of the acetabulum in the aOP‐based sCT

The value of ${\bar{\bar{{\text{MAE}}_{\text{bone}}}}}^{\text{RS}}\left(\text{aOP}\right)$ was higher than ${\bar{\bar{{\text{MAE}}_{\text{bone}}}}}^{\text{RS}}\left(\text{aIP}\right)$ by up to 28% on the canine data set and by up to 17% on the human data set. The value of ${\bar{\bar{\text{DSC}}}}^{\text{RS}}$ varied by up to 0.05 units on both data sets. This difference is visually supported by the sCT‐to‐CT absolute difference maps presented in Figure 6. Contrary to soft‐tissue reconstructions for which error maps are noise‐like (Supporting Information Figure S1), bone‐reconstruction difference maps clearly depict bone structures. However, the distribution of errors is model‐dependent. Errors appeared primarily in the bone marrow for the aIP configuration (Figures 5B and 6A) and in the cortical bone for the aOP configuration. More specifically, cortical bone intensity was either hypo‐intense (pelvis in Figures 5A and 6A) or hyperintense (femoral heads in Figure 5B and acetabulum in Figure 6B), resulting in very high MAE_bone_ for the aOP configuration (Table 1).

Figures 5C and 6B also present a typical case of systematic errors in regions such as the bowels, which are caused by the lack of correspondence between MR and CT. These errors cannot be corrected by registration alone, as they are due to physiological changes between scanning sessions.

**Figure 7:**
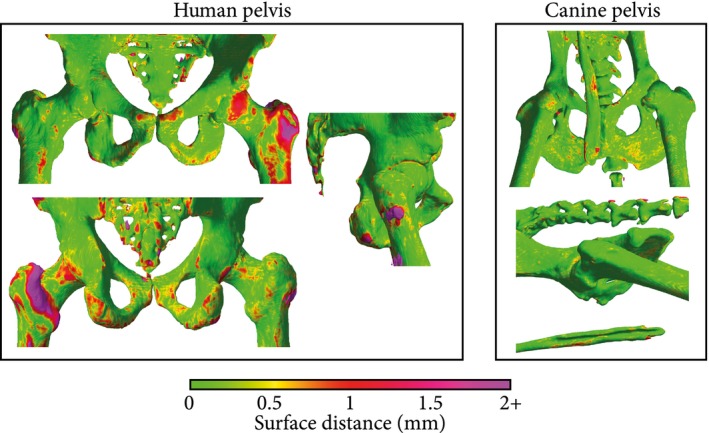
Surface distance maps obtained for human (subject 8) (A) and canine (subject 13) (B) subjects. Meshes were obtained by thresholding the CT at 200 HU and removing unconnected components. The color map indicates the bilateral surface distance between the CT and a sCT obtained using a Dixon input configuration. The high errors in the human subject, especially in the left femur, originate from registration errors between the CT and the MR/sCT. The canine bone rendering shows the baculum, a thin penile bone present only in a small fraction of the data set that resulted in higher errors for male subjects

Figure 7 shows 3D bone renderings mapped with bilateral sCT‐to‐CT surface distance obtained for human and canine subjects. Locally, the largest errors appeared in the transverse process of vertebrae, in the femur, and in the sacral bone. These errors were sometimes dominated by registration errors, especially in the in vivo human subjects (Figure 7A). However, the surface distance was on average submillimeter, as shown in Table 1, which suggests an overall accurate pelvic geometry. The aOP‐models had the highest errors, with mean errors up to 0.6 mm, but no significant difference was seen among all other models.

## 4 DISCUSSION

In this study, we compared 6 input configurations. For each input configuration, a deep learning–based model was trained up to 10 times for sCT generation in pelvic scans of ex vivo canines and human prostate cancer patients. Input configurations contained 1, 2, or 4 MR images obtained from a single T_1w_‐MGE sequence. Each configuration incorporated diverse information content related to proton density, $T_{2}^{*}$ relaxation, and water and fat content, as determined by the TEs and presence or absence of Dixon reconstructions.

This study showed that the choice of MR images within an input influenced sCT generation models in 3 ways. First, the aIP input configuration outperformed the aOP configuration. This is most likely related to the lower degree of correlation between MR and CT intensities (Figure 3) in OP images. The OP images present destructive interferences between water and fat protons, resulting in signal voids in regions at the interface between fat and muscle. These interferences are not present in IP images, rendering IP images favorable for sCT generation. This result is in accordance with the use of ZTE images for sCT generation,^8^, ^10^, ^29^ which also present a strong correlation between MR signal and cortical bone.^18^, ^21^ However, a study by Leynes and Larson^32^ showed that Dixon‐reconstructed IP images outperformed ZTE images in sCT generation tasks. Another aspect that may have played a role in the performance difference observed between aIP and aOP images is their amount of $T_{2}^{*}$ weighting. However, aIP models outperformed aOP models in both canine (1.5 T) and human (3 T) data sets, although their relative amount of $T_{2}^{*}$ weighting differed due to their different TEs. The $T_{2}^{*}$ weighting in the aIP echo was high in the canines and low in the humans when compared with the $T_{2}^{*}$ weighting in the aOP echo. Hence, the effect of $T_{2}^{*}$ decay appeared less important than the interference between water and fat protons at the considered TEs. Consequently, the performance and repeatability of a model based on single‐channel T1_w_‐MGE inputs were dependent primarily on the water–fat proton dephasing associated with the choice of TE during the acquisition. The ability of the IP configuration to accurately reconstruct cortical bone, which is crucial in a CT contrast, suggests the importance of IP images for sCT generation.

Second, presenting multiple channels as inputs to the model improved sCT generation as compared with single‐channel models. In particular, the WF configurations outperformed the in‐phase channel despite their close linear relation. This result suggests that explicit water–fat decomposition is favorable and important to the model. In addition, the Dual input configuration containing both aIP and aOP images outperformed separately trained aIP and aOP models. Because the complexities of the single‐channel and dual‐channel models were virtually identical, this benefit resulted from the combined use of the information present in the separate images. Considering these 2 results, we can hypothesize that IPOP and Dual were potentially able to access information similar to water–fat decomposition.

Third, within multichannel models, the Dixon input configuration outperformed the others despite containing interdependent MR images. Presumably, explicitly providing this additional information simplified the learning task, improving processes such as soft‐tissue discrimination. Note that this result does not imply that more data in the input necessarily results in better performance.^32^ Furthermore, models based on both IP and OP images, either acquired (Dual) or reconstructed (IPOP), performed similarly. Likewise, no statistically significant difference was found between the aIP and IP input configurations, as shown in Supporting Information Table S2. Hence, clinically, a standard multi‐echo gradient‐echo sequence can be acquired with flexibility in TE for favorable soft‐tissue visualization, and the available water/fat‐separated reconstructions can be used for sCT generation. All of these differences between input configurations were more apparent in the canine data set, which was more challenging due to its variability. This result suggests that using multichannel inputs can increase the robustness of a model.

Aiming for a fair comparison between the different input configurations, we chose to keep the same architecture for the different models. The resulting architecture may have been suboptimal for multichannel input processing, but demonstrated significantly better results for multichannel input configurations. Taking this fourth dimension into account in the design of the network, such as by considering separate paths per channel or 4D convolutions, could have potentially further amplified the difference between single and multichannel input configurations but at the cost of increased model complexity.

The differences observed between the different models were reinforced by the repeatability experiment we performed. It established a SD as low as 1 HU for most models, which proves that the input‐related differences in this study were statistically significant. Besides, the systematic improvements seen on aIP over aOP and multichannel over single‐channel input configurations justify a prevailing use of IP and Dixon reconstructed images.

Quantitative evaluation on the human data set showed that, depending on the models, ${\bar{\bar{{\text{MAE}}_{\text{body}}}}}^{\text{RS}}$, the MAE_body_ averaged across replicates and subjects, varied between 32.1 HU and 40.0 HU. Similarly, ${\bar{\bar{\text{ME}}}}^{\text{RS}}$ ranged from 0.0 HU to −2.0 HU and σ^ME^ from 4.3 HU to 9.2 HU. The value of ${\bar{\bar{\text{ME}}}}^{\text{RS}}$ obtained for aOP models were desirably centered at 0.0 HU, but they displayed a high SD, suggesting their low precision, which is unfavorable for clinical adoption.

Overall, the models we trained show competitive quantitative results when compared with the related literature on deep learning–based sCT generation in pelvic anatomy. Using single MR images, Nie et al^31^ obtained a MAE_body_ of 42.4 ± 5.1 HU and a PSNR of 33.4 ± 1.1 dB with a 3D fully convolutional neural network. Using Dixon reconstructed inputs, Maspero et al^6^ obtained a MAE of 61 ± 9 HU and a ME of 2 ± 8 HU using a 2D conditional generative adversarial network. With a 3D UNet trained on Dixon and ZTE images (fractional fat, fractional water), Leynes et al^10^ reported a ME of −12 ± 79 HU. Although all of these studies were based on deep learning approaches and were performed on the same anatomy, any comparison is difficult, as the CT scans differ in slice spacing and noise levels, and the workflows differ in terms of preprocessing methods (rigid or nonrigid registration, air pockets relocation on the CT) in the architecture of the neural network and in the spatial dimensionality of their input. Such variations can even occur within a study when different data sets are used. Accordingly, the variations we observed between the human and canine data sets are probably related to interpatient anatomical variability as well as to differences in acquisition parameters such as CT slice thickness. However, within 1 data set, the models demonstrated the impact of choices made about the MR input configurations, both in terms of performance and interpatient variability.

As reported in previous studies,^27^, ^30^ the largest errors occurred in the bone anatomy. The results we obtained for the bone (MAE_bone_ of 114 ± 56 HU and DSC_bone_ of 0.83 ± 0.11) are in accordance with Fu et al,^30^ who reported a MAE_bone_ of 154.3 ± 22.3 HU and a DSC_bone_ of 0.82 ± 0.04 in 20 prostate cancer patients using a 3D convolution neural network. However, we showed that bone reconstruction (voxels > 200 HU) was also input‐dependent, with differences in ${\bar{\bar{{\text{MAE}}_{\text{bone}}}}}^{\text{RS}}$ of up to 28% between aOP and aIP input configurations.

Registration errors related to interscan motion, physiological changes, and deformations inherent to MRI generated noise during the training and introduced an offset in the evaluated metrics, especially in bone structures. To limit their influence, which could potentially have concealed model‐related differences, we applied nonrigid CT‐to‐MR registration on our data sets. In addition, because we used a single sequence for MR image acquisition, all images within an input were perfectly registered. Therefore, all input configurations experienced the same MR‐to‐CT registration errors, and multichannel models were not corrupted by registration errors between the channels, as can be the case with Dixon‐ZTE combinations.

In this work, we demonstrated that the choice of acquisition parameters, and consequently MR contrasts, strongly influences the performance and repeatability of deep learning–based sCT generation models. This study does not aspire to determine the optimal MR input for sCT generation, as it may vary with anatomies and applications. For instance, for radiotherapy treatment planning purposes, inputs that do not lead to classification errors would be favored. For orthopedics, getting an accurate bone geometry and capturing variations in HU within the bone are required. However, the observed high repeatability and significant differences found between input channels justify the search for that optimal input.

## 5 CONCLUSIONS

We studied the influence of gradient echo–based contrasts as input to deep learning–based sCT generation models in canine and human populations. Two parameters were found to influence the performance and repeatability of sCT generation. First, the TE‐related water–fat interference of single MR images affected the performance of a model. Overall, in‐phase images outperformed opposed‐phase images because of their higher bone specificity. Second, the use of multiple related MR images combined in the input as channels improved the performance and robustness of a model. In particular, the Dixon input configuration showed the best results in terms of performance and repeatability, although it contained interdependent MR images.

This study established the influence of the choices made during MR acquisition for sCT generation. The systematic and statistically significant improvement that was demonstrated motivates the research of an optimal MR contrast or combination of MR contrasts for a given task.

## CONFLICT OF INTEREST

M. van Stralen and P.R. Seevinck are minority shareholders at MRIguidance B.V.

## Supporting information


**FIGURE S1** Comparison of sCTs generated by the input configurations for a canine subject with a focus on soft tissues. The Dual input configuration was omitted, as it was very similar to IPOP. A region defined by the red square was enlarged and its window level adapted to highlight soft tissue. Errors maps show the absolute errors between the sCTs and the CT
**TABLE S1** Structural similarity index (± σ) obtained for each model per data set, averaged across replicates and subjects
**TABLE S2** Quantitative comparison between the aIP echo acquired from the T1w‐MGE sequence and the corresponding Dixon‐reconstructed in‐phase image (measurements were averaged across replicates and subjects [± σ])
