# Supplementary material for: Deep learning–based MR‐to‐CT synthesis: The influence of varying gradient echo–based MR images as input channels
Source: Magn Reson Med. 2019 Oct 8;83(4):1429–41. doi: 10.1002/mrm.28008 (PMC6972695; doi:10.1002/mrm.28008)
Supplement: Supplementary file 1 — FIGURE S1 Comparison of sCTs generated by the input configurations for a canine subject with a focus on soft tissues. The Dual input configuration was omitted, as it was very similar to IPOP. A region defined by the red square was enlarged and its window level adapted to highlight soft tissue. Errors maps show the absolute errors between the sCTs and the CT TABLE S1 Structural similarity index (± σ) obtained for each model per data set, averaged across replicates and subjects TABLE S2 Quantitative comparison between the aIP echo acquired from the T1w‐MGE sequence and the corresponding Dixon‐reconstructed in‐phase image (measurements were averaged across replicates and subjects [± σ]) [file MRM-83-1429-s001.docx]

| Pop. | Input | SSIM |
| --- | --- | --- |
| Human | aIP | 0.74 ± 0.05 |
|  | aOP | 0.73 ± 0.05 |
|  | Dual | 0.75 ± 0.05 |
|  | IPOP | 0.75 ± 0.05 |
|  | WF | 0.75 ± 0.05 |
|  | Dixon | 0.75 ± 0.05 |
| Canine | aIP | 0.84 ± 0.07 |
|  | aOP | 0.83 ± 0.07 |
|  | Dual | 0.85 ± 0.07 |
|  | IPOP | 0.85 ± 0.07 |
|  | WF | 0.85 ± 0.07 |
|  | Dixon | 0.85 ± 0.07 |

Table S1: Structural similarity index ($\pm\sigma$) obtained for each model per dataset averaged across replicates and subjects.


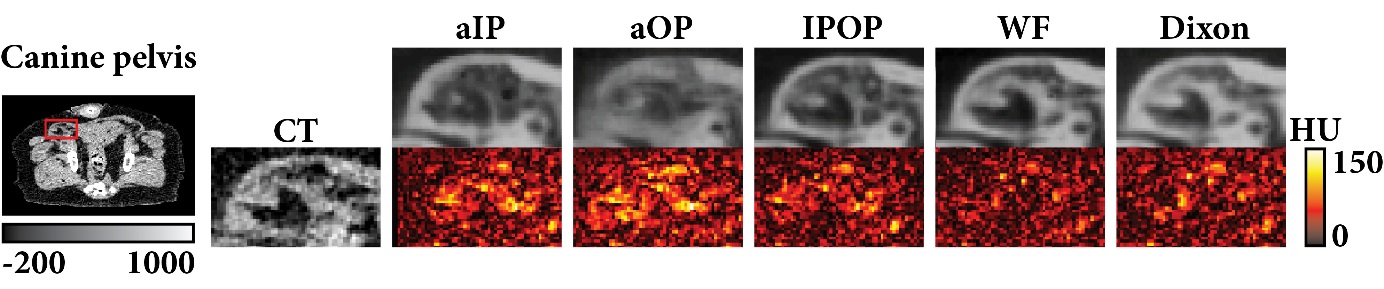


Figure S1: Comparison of sCTs generated by the input configurations for a canine subject with a focus on soft tissues. The Dual input configuration was omitted as it was very similar to IPOP. A region defined by the red square was enlarged and its window level adapted to highlight soft tissue. Errors maps show the absolute errors between the sCTs and the CT.

| Pop. | Input | MAE | MAE_bone_ | DSC_bone_ | PSNR | SSIM |
| --- | --- | --- | --- | --- | --- | --- |
| Human | aIP | 34.1 ± 7.8 | 123 ± 54 | 0.81 ± 0.11 | 36.1 ± 2.3 | 0.74 ± 0.05 |
|  | IP | 34.6 ± 8.3 | 121 ± 55 | 0.82 ± 0.10 | 36.0 ± 2.3 | 0.74 ± 0.05 |
| Canine | aIP | 42.2 ± 8.3 | 144 ± 42 | 0.91 ± 0.03 | 35.1 ± 1.6 | 0.84 ± 0.07 |
|  | IP | 41.4 ± 5.8 | 143 ± 35 | 0.91 ± 0.03 | 35.1 ± 1.7 | 0.84 ± 0.07 |

Table S2: Quantitative comparison between the almost in-phase echo acquired from the T1w-MGE sequence and the corresponding Dixon reconstructed in-phase image. Measurements were averaged across replicates and subjects (± σ).
